# Twenty-year evolution of *Leishmania infantum* infection in dogs in Valdeorras (Galicia, Northwestern Spain): implication of climatic factors and preventive measures

**DOI:** 10.1186/s13071-024-06357-8

**Published:** 2024-07-01

**Authors:** Patricia Olmeda, David Díaz-Regañón, Alejandra Villaescusa, Inmaculada Amusategui, Adolfo García, Francisco Herrero, Miguel A. Tesouro, Fernando Rodríguez-Franco, Mercedes García-Sancho, Daniel Martín-Fraile, Ángel Sainz

**Affiliations:** 1 Department of Animal Medicine and Surgery, College of Veterinary Medicine, Complutense University of Madrid, Avda. Puerta de Hierro S/N, 28040 Madrid, Spain; 2 Veterinary Clinic “Servicios Veterinarios del Sil”, C/ Coruña 9, O Barco de Valdeorras, 32300 Ourense, Spain; 3 Department of Veterinary Medicine, Surgery and Anatomy, College of Veterinary Medicine, University of León, Campus de Vegazana, 24071 León, Spain

**Keywords:** Canine leishmaniosis, Immunofluorescence antibody test (IFAT), *Leishmania infantum*, Prophylaxis, Climate change, *Phlebotomus*

## Abstract

**Background:**

Abiotic factors play a significant role in the evolution of *Leishmania infantum* infection due to its vectorial nature. This study aims to assess the evolution in the detection of new *L. infantum* infection cases in Valdeorras (Ourense, Northwestern Spain) over a 20-year period and how different climatic variables and preventive measures may have affected it.

**Methods:**

Indirect immunofluorescence antibody tests (IFAT) were performed on serum samples collected from dogs attending the ‘Servicios Veterinarios de Sil’ veterinary clinic (Valdeorras, Northwestern Spain) between May 2003 and April 2023 to detect *L. infantum* exposure. The percentage of new cases of *L. infantum* infection was calculated from May of one year to April of the following year. Climatic conditions in the region, global sales of ectoparasiticides and the number of vaccines against *L. infantum* delivered in the veterinary clinic from 2003 to 2022 were recorded. Statistical analyses were conducted to determine the associations between these factors and the percentage of new cases of *L. infantum* infection.

**Results:**

A total of 2909 dogs were assessed, and 3785 IFAT tests were performed between May 2003 and April 2023. The mean percentage of new seropositive cases over the 20-year period studied was 21.65 ± 10.8%, with a decline from the beginning to the end of the period studied. The percentage was significantly higher between May 2003 and April 2008 compared with the other periods (May 2008 to April 2013, May 2013 to April 2018 and May 2018 to April 2023). There was a positive correlation between the percentage of new cases of *L. infantum* infection and the maximum relative humidity in winter. Conversely, there was a negative correlation between the percentage of new cases and sales of ectoparasiticides and vaccination against *L. infantum*.

**Conclusions:**

This study is one of the longest evaluations of the evolution of *L. infantum* infection in a fixed location and its association with external factors including climatic conditions and preventive measures. The results confirm that Valdeorras is a high-risk area for *L. infantum* infection. The use of ectoparasiticides and vaccines against *L. infantum* has been shown to play a significant role in preventing *L. infantum* infection, highlighting the crucial role of veterinarians in the fight against this disease.

**Graphical Abstract:**

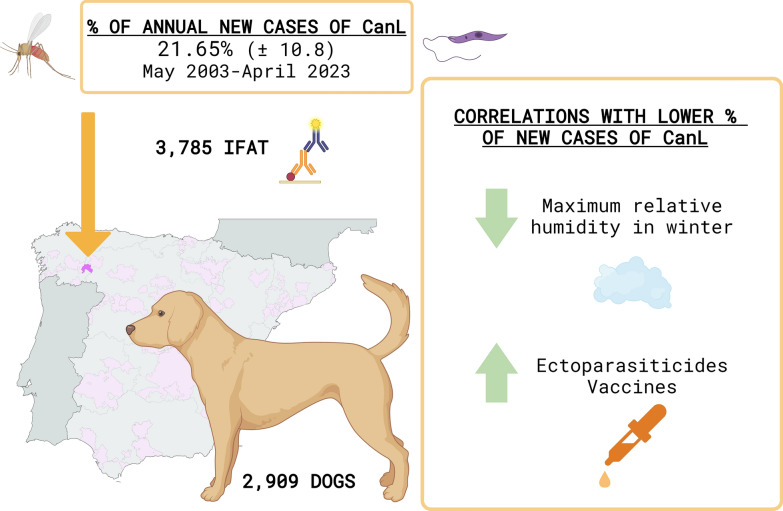

## Background

Canine leishmaniosis (CanL) is a disease caused in Spain by the protozoan *Leishmania infantum* [1, 2]. Dogs constitute the main reservoir for this parasite [1–4]. Although occasional vertical and horizontal transmission has been described [2–5], the main mode of transmission in Europe is the bite of an infected female phlebotomine sand fly (*Phlebotomus perniciosus* and *P. ariasi*) [3, 4, 6]. Traditionally, the geographical distribution of phlebotomine sand flies had been estimated to be between 50° north and 40° south latitudes [3, 7]. However, as predicted by some authors, the distribution has expanded beyond these latitudes [8–12].

In this context, Northwestern Spain was traditionally considered a leishmaniosis-free region, presumably because its cold and rainy climate limits the presence of sand flies [13, 14]. Nevertheless, two cases of apparently autochthonous canine leishmaniosis were reported in Galicia in the early 1990s [15]. In addition, a seroprevalence study carried out in the early 2000s in Valdeorras (Ourense, Galicia, Spain) changed the perception of the disease in this area, being henceforth considered hyperendemic for *L. infantum* infection [13]. The prevalence of *L. infantum* infection reported in Galicia ranges from 2.5% to 7.5% [13, 14, 16], while in Ourense it has been found to be higher, from 7.5% to 35.6% depending of the studies and their methodology [5, 13, 17]. Ourense presents a particular supra-Mediterranean climate, which distinguishes it from other nearby regions in northern Spain belonging to the temperate bioclimatic zone, where the prevalence of *L. infantum* infection is lower [5].

The main phlebotomine species vectors of *L. infantum* described in these areas are *Phlebotomus perniciosus* and *P. ariasi* [5, 18–20]. *Phlebotomus ariasi* is less abundant and is found in more localised regions due to its preference for colder, more humid and mountainous climates, as well as for higher altitudes [5, 7, 9, 18, 21–24]. *Phlebotomus perniciosus* is more widespread because it is less affected by climatic conditions. It prefers drier and warmer environments and is found at lower altitudes than *P. ariasi* [5, 9, 18, 25]. Both *P. perniciosus* and *P. ariasi* have been previously reported in Ourense Province [5, 19]. It is considered that the phlebotomine sand fly season in Spain begins in early May, although in some regions it has been reported as early as April, and ends between late October and November, with its peak activity between July and September [3, 4, 26–28]. It is determined by the climatic conditions of the region, such as temperature, relative humidity and wind speed [20, 29]. Any change in the environment alters the ecological balance and the context in which parasites and vectors reproduce and transmit disease [30, 31].

The main measures for the prevention of *L. infantum* infection include the use of ectoparasiticides as the most effective strategy, accompanied by the vaccination of healthy seronegative dogs against *L. infantum* to prevent the development of the disease and reduce the risk of transmission [4, 32, 33].

The aim of this study was to assess the evolution of the percentage of annual new cases of *L. infantum* infection over a 20-year period (2003–2023) in a specific area with a known high prevalence of the parasite (Valdeorras, Ourense, Northwestern Spain). Furthermore, considering the multitude of factors influencing the presence of *L. infantum* infection in a given area, we aimed to determine the possible influence of certain climatic variables and preventive measures implemented over the time on this *L. infantum* infection incidence. We hypothesised that the percentage of newly detected cases in this area has decreased over the 20-year period. We further suggested that climatic variables that potentially could impact the vector and its habitat, along with implemented preventive measures, are interrelated factors that could potentially influence the incidence of *L. infantum* infection.

## Methods

### Study design

This retrospective longitudinal study was conducted in O Barco de Valdeorras, Ourense, Galicia (Northwest Spain: 42° 25′ 0″ N, 6° 58′ 59″ W), which occupies a portion of the alluvial plain of the Sil River, nestled amidst the surrounding mountain ranges. It represents a narrow and deep tectonic trench, along which the river follows the course dictated by a network of fractures. The municipality’s altitude ranges from 310 to 1537 m, being the village at an elevation of 326 m above sea level. It hosts a population of 13,277 inhabitants, with no significant variations since 2003 up to the present day. All the samples were collected at the ‘Servicios veterinarios de Sil’ veterinary clinic over a 20-year period (from May 2003 to April 2023). Four practices are currently working in O Barco de Valdeorras, but this clinic was the first to be open in the municipality.

The study included dogs tested for *L. infantum* infection by indirect immunofluorescence antibody test (IFAT) based on practitioners’ clinical criteria, including prophylactic health check-up plans, regardless the presence or absence of clinical signs compatible with CanL. Practitioners responsible for the recruitment of samples were the same during the entire period of study. Dogs that had been vaccinated against leishmaniosis or that had previously tested positive were excluded.

### IFAT serodiagnosis

Serum of canine blood samples (25 µL) was used for diagnosis at the Canine Leishmaniosis and Ehrlichiosis Diagnostic Service of the Complutense Veterinary Teaching Hospital of the Complutense University of Madrid. Serodiagnosis was performed by detecting specific antibodies against *L. infantum* using the indirect immunofluorescence antibody test (IFAT) for anti-*Leishmania*-specific immunoglobulin G (IgG) antibodies as previously described [34–36]. Antigen was obtained from a culture of promastigotes of *L. infantum* L-75 established in Novi, McNeal and Nicolle medium. Plates were examined using an Olympus BH-2® epifluorescence microscope (Olympus Imaging America Inc., Center Valley, Pennsylvania, United States) with a blue filter and ×400 objective.

### Serodiagnosis data collection

Each sample was identified with a laboratory registration number and was blindly analysed. The IFAT result of each sample was classified as positive (≥ 1/100) or negative (< 1/100), considering the established cut-off (1/100).

The percentage of new cases of *L. infantum* infection was calculated for 12-month periods (total of 20 periods) from May of one year to April of the following year, corresponding to the sand fly season. For the analysis comparison, the data were grouped in four clusters: May 2003 to April 2008, May 2008 to April 2013, May 2013 to April 2018 and May 2018 to April 2023.

### Climatic variables

The dataset utilised in this study compromises daily measurements of precipitation (mm), temperature (°C) and relative humidity (%) spanning the period from 2003 to 2022 in Valdeorras. These data were sourced from the Spanish Meteorological Agency (AEMET), considering the official data provided by the meteorological observatory located in O Barco de Valdeorras. This observatory was located 650 m away from the veterinary clinic where this study was performed. Parameters including mean, maximum and minimum values for temperature, total precipitation, and maximum and minimum relative humidity were recorded for each season: spring (March–May), summer (June–August), autumn (September–November) and winter (December–February).

Furthermore, aggregate metrics were computed, encompassing total annual precipitation, precipitation of the wettest month and season, precipitation of the driest season, precipitation of the warmest and coolest seasons, mean temperatures of the warmest and coolest seasons, and the lowest and mean minimum temperatures for the month of July for each year within the study period. These variables have been previously reported to potentially influence the development of *Phlebotomus* or the prevalence of canine leishmaniosis [8, 20, 23, 28, 37, 38].

### Ectoparasiticides and vaccinations against canine leishmaniosis

Data were collected from the overall annual sales of ectoparasiticides and the annual number of vaccinations against canine leishmaniosis administered at the ‘Servicios veterinarios del Sil’’ veterinary clinic during the study period (2003–2022).

### Statistical analysis

The results were statistically analysed using the IBM SPSS Statistics software v.29.0.2.0 (IBM, Armonk, NY, USA). Significant differences among the percentages of new positive dogs for *L. infantum* infection in the different periods of time were analysed using the chi-square test. As the Shapiro–Wilk test indicated a lack of normal distribution, a Spearman’s correlation analysis was employed to evaluate the relationship between the percentage of new positive cases and climatic variables. Considering the sand fly season and the time needed for *L. infantum* antibody production after infection, for seasonal variables, data were collected from winter and spring from the same year and from summer and autumn from the previous year [20, 23, 26]. Additionally, sales of ectoparasiticides in dogs and vaccinations against canine leishmaniosis were also assessed using Spearman’s correlation test. The linear trend of the variables over time was analysed and graphically represented using GraphPad Prism 9.4 software (GraphPad Software, Boston, Massachusetts, USA). A statistically significant difference was set at *P* < 0.05.

## Results

A total of 2909 dogs met the inclusion criteria during this period. Globally, a total of 3785 IFAT tests were conducted. There are more IFATs than dogs because some of them had the test performed several times over their life during the 20-year period of study.

**Fig. 1:**
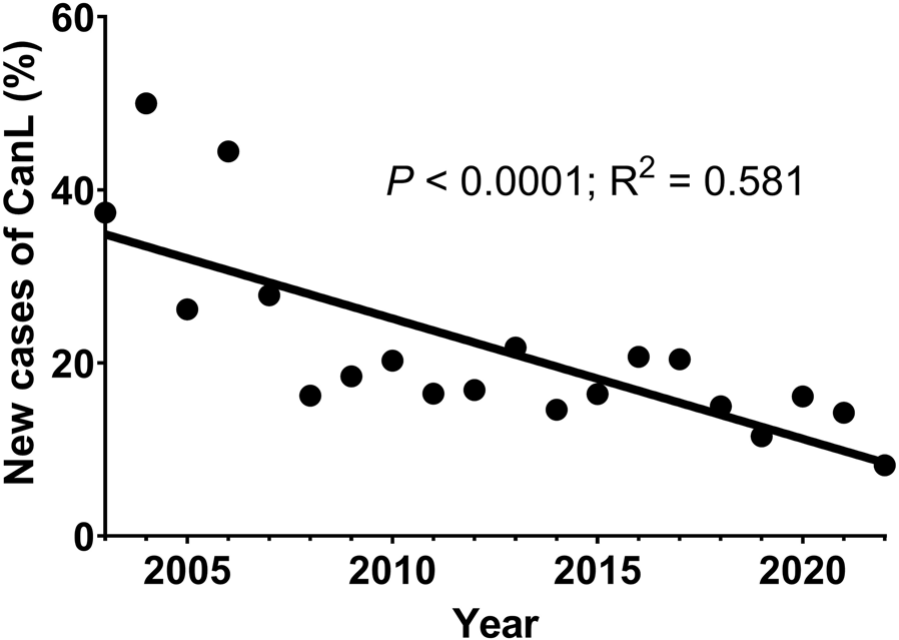
Annual percentage of new *L. infantum* infection cases. Trend over phlebotomine sand fly activity seasons. The line represents the linear trend (*P* < 0.0001, *R*^*2*^ = 0.581), indicating a decreasing trend. Each data point corresponds to the percentage (%) of new *L. infantum* infection cases diagnosed annually, using the period season of the phlebotomine sand fly activity

The percentage of new *L. infantum* infection cases was calculated for each sand fly season, as outlined in Table 1. The mean percentage of new seropositive cases over the 20-year period studied was 21.65 ± 10.8%. These percentages, classified into grouped clusters were as follows: 35.3% (May 2003 to April 2008), 17.9% (May 2008 to April 2013), 18.8% (May 2013 to April 2018) and 12.9% (May 2018 to April 2023). The results obtained in the first cluster (May 2003 to April 2008) were significantly higher than those of subsequent groups, while the fourth cluster (May 2018 to April 2023) showed significant lower values (*χ*^2^ = 112.553, *df* = 3, *P* < 0.001), indicating a decreasing linear trend as illustrated in Fig. 1.

**Table 1:** Percentage of new *L. infantum* infection cases in the sandfly seasons from May 2003 to April 2023

Sandfly season	Number of samples tested	Positive (≥ 100)	Negative (< 100)	Percentage of new cases (%)
2003/04	107	40	67	37.38
2004/05	60	30	30	50.00
2005/06	84	22	62	26.19
2006/07	72	32	40	44.44
2007/08	133	37	96	27.82
2008/09	111	18	93	16.22
2009/10	233	43	190	18.45
2010/11	237	48	189	20.25
2011/12	231	38	193	16.45
2012/13	166	28	138	16.87
2013/14	248	54	194	21.77
2014/15	233	34	199	14.59
2015/16	189	31	158	16.40
2016/17	145	30	115	20.69
2017/18	235	48	187	20.43
2018/19	267	40	227	14.98
2019/20	278	32	246	11.51
2020/21	248	40	208	16.13
2021/22	239	34	205	14.23
2022/23	269	22	247	8.18
Total	3785	701	3084	21.65

Positive (≥100), number of samples with an IFAT title ≥1/100; Negative (<100), number of samples with an IFAT title <1/100. Sandfly seasons comprehend from May of the first year to April of the following year

Seasonal precipitation, temperature and relative humidity in Valdeorras from 2003 to 2022 are presented in Tables 2 and 3. The wettest season varied from year to year among spring, autumn and winter. Based on the series of data available, the wettest year was 2016 (1019.5 mm), with an especially rainy winter (550.7 mm), while the driest year was 2004 (344.4 mm) (Table 2). The driest season was mainly summer, except for the autumn of 2007, spring of 2009 and winter of 2012 (Table 3). The lowest total precipitation recorded during the driest season of all the years with available data was 35.2 mm in summer of 2008. The warmest season recorded was always summer, with the highest mean temperature of 24.3 °C in 2022. Conversely, the summer’s lowest mean temperature was 20.8 °C in 2007. Winter was consistently the coldest season, with the highest mean temperature of 8.7 °C in both 2016 and 2020. The lowest minimum temperature in July over the 20-year period of study, was recorded in 2012 at 7.5 °C. Additionally, the lowest mean minimum temperature in July was recorded in 2009 at 13.9 °C.

**Table 2:** Seasonal precipitation, temperature and relative humidity in Valdeorras from 2003 to 2022

Year			2003	2004	2005	2006	2007	2008	2009	2010	2011	2012	2013	2014	2015	2016	2017	2018	2019	2020	2021	2022
Precipitations (mm)	Total	W			97.4	200.8	213.0	211.6	286.2	413.2		61.0	424.6		255.2	550.7	191.8		168.8	178.0	432.0	119.4
		Sp			170.4	146.6	125.4	296.2	93.4	132.4	155.2	146.6	347.3	125.6	126.2	387.2	232.2	197.2	165.8	124.6	61.2	87.6
		S			78.6	115.8	112.8	35.2	94.6	80.6	41.2	69.6	98.0	53.6	50.6	48.8	43.6	144.0	141.2	92.4	37.0	54.6
		A		201.4	129.4	303.2	44.6	74.4	251.8		132.6	198.2		296.0	253.5	58.2	83.0	217.6	294.2	207.4	160.2	154.6
	Total annual			344.4	515.8	752.6	439.8	688.8	837.2			573.6		678.8	704.9	1019.5	661.8		873.4	668.2	605	415.6
Temperature (ºC)	Mean	W	7.1	7.8	4.9	5.1	7.6	7.6	6.6	6.6	6.7	6	7.5	7.5	6	8.7	7.2	7	7	8.7	7.9	8
		Sp	14.4	12.5	13.9	15.2	14	13.1	13.8	14.1	15.4	13.9	12.6	13.9	14.9	12.7	15.5	13.2	14	15.6	14	14.5
		S	23.7	22.6	23.7	23.6	20.8	21.3	22.1	23.1	21.6	21.4	22.7	21.3	23.4	23.1	23.4	22.4	21.7	22.5	21.8	24.3
		A	14.6	14.2	14.8	16.6	14.2	13.3	16.4	14.3	15.6	14.8	14.9	16	14.9	14.7	14.8	15.6	15.3	15.3	14.2	16.4
	Maximum	W	10.5	11.6	9.6	9.6	11.7	12.1	10.7	10.3	10.8	10.9	11	11	9.9	12.4	11.7	10.8	11.7	12.7	11.6	13.1
		Sp	20.7	18.8	20.6	21.6	20.6	18.6	21.4	20.7	22.4	20.4	18.2	19.7	21.9	18.2	22.7	18.9	21.1	21.8	20.6	21
		S	30.7	30	32	31.8	28	28.8	29.7	31.3	29.2	28.7	30.9	28.7	31.3	31.1	30.9	29.1	28.9	29.9	29.1	32
		A	19.6	20	20.9	21.8	21.6	18.8	22.1	19.7	21.9	20.5	20.4	20.8	20.3	21.1	21.7	21.4	20.3	20.7	19.7	21.6
	Minimum	W	3.8	4.1	0.1	0.5	3.5	3	2.5	3	2.6	1.1	4	3.9	2.1	5	2.6	3.2	2.2	4.7	4.2	2.8
		Sp	8.1	6.3	7.3	8.8	7.4	7.5	6.1	7.6	8.5	7.4	7.1	8.1	8	7.2	8.3	7.5	6.9	9.4	7.4	8
		S	16.7	15.3	15.4	15.5	13.5	13.9	14.4	14.9	14	14.1	14.6	14	15.6	15.2	15.8	15.7	14.5	15.1	14.6	16.6
		A	9.7	8.3	8.8	11.3	6.7	7.7	10.6	8.9	9.3	9.1	9.4	11.3	9.5	8.4	7.9	9.7	10.3	9.9	8.7	11.1
Relative humidity (%)	Maximum	W							95.8	95.6	94.9	93.5	95.5	93.7	95.2	94.5	94.3	95.2	94.3	92.4	93.6	92.5
		Sp							88.8	87.5	90.3	86	87.9	88.4	86.2	90.3	84.4	88	85.7	88.4	87.2	84.6
		S							81.9	84.5	82.8	83.1	82.7	84.3	80.8	82.8	80	85.1	84	82.9	83.2	81.2
		A						93	90.9	93	90.1	90.5	91.7	92.8	92.2	89.8	88.6	89.6	89.8	89.6	92.1	89.1
	Minimum	W							61.2	58.5	59.5	58	63.6	61.3	64.1	62.8	60.6	66.1	60.7	60.5	63.6	55.1
		Sp							33.5	35.5	38	36	40.8	41.7	34.4	42.5	33.1	40.7	34.4	40.8	36.8	37.4
		S							28.4	30	30.4	31.9	27.5	32.1	28.4	30.4	29.7	37.8	34.6	33.3	33	29.4
		A						50	45.9	49.4	43.8	46.8	49.3	54	51.6	43.9	38.9	46.8	51.3	49.6	51.3	47.3

W, winter; Sp, spring, S, summer, A, autumn. Blank spaces indicate missing data during certain periods due to the retrospective nature of the study. The data were obtained from the Spanish Meteorological Agency (AEMET), and aggregate metrics were calculated accordingly

**Table 3:** Selected climate characteristics in Valdeorras from 2003 to 2022

Year	Wettest month		Wettest season		Driest season		Warmest season			Coldest season			Temp July	
	Month	Prec	Season	Prec	Season	Prec	Season	Prec	Temp	Season	Prec	Temp	Lowest min temp	Mean min temp
2003							S		23.7	W		7.1	11.0	15.5
2004							S		22.6	W		7.8		
2005	12	96	Sp	170.4	S	78.6	S	78.6	23.7	W	97.4	4.9	10.6	16.1
2006	10	161.6	A	303.2	S	115.8	S	115.8	23.6	W	200.8	5.1	12.2	17.2
2007	2	110.6	W	213	A	44.6	S	112.8	20.8	W	213.0	7.6	9.6	14.2
2008	1	114.8	Sp	296.2	S	35.2	S	35.2	21.3	W	211.6	7.6	8.5	14.4
2009	12	208.8	W	286.2	Sp	93.4	S	94.6	22.1	W	286.2	6.6	8.6	13.9
2010							S	80.6	23.1	W	413.2	6.6	10.3	16.1
2011							S	41.2	21.6	W		6.7	9.5	14.1
2012	12	139.6	A	198.2	W	61	S	69.6	21.4	W	61.0	6.0	7.5	14.2
2013							S	98.0	22.7	W	424.6	7.5	12.8	16.8
2014	11	162					S	53.6	21.3	W		7.5	10.5	15.3
2015	10	147.4	W	255.2	S	50.6	S	50.6	23.4	W	255.2	6.0	13.7	16.9
2016	1	280.1	W	550.7	S	48.8	S	48.8	23.1	W	550.7	8.7	10.3	16.0
2017	5	158.4	Sp	232.2	S	43.6	S	43.6	23.4	W	191.8	7.2	8.7	16.0
2018							S	144.0	22.4	W		7.0	13.0	16.7
2019	11	158.8	A	294.2	S	141.2	S	141.2	21.7	W	168.8	7.0	11.2	16.1
2020	12	184.4	A	207.4	S	92.4	S	92.4	22.5	W	178.0	8.7	9.9	16.9
2021	2	142.8	W	432	S	37	S	37.0	21.8	W	432.0	7.9	10.1	15.0
2022	11	99.6	A	154.6	S	54.6	S	54.6	24.3	W	119.4	8.0	9.2	17.5

Month, number of the month (1–12); Prec, precipitation (mm); Temp, temperature (°C); Temp July, temperature of the month of July; Lowest min temp, lowest minimum temperature (°C); Mean min temp, mean minimum temperature (°C); W, winter; Sp, spring, S, summer, A, autumn

Blank spaces indicate lack of information in that period of time due to the retrospective nature of the study

Table 4 presents the overall sales of ectoparasiticides and vaccinations against *L. infantum* infection in the clinic. From 2003 to 2022, a total of 3702 ectoparasiticides were sold in the ‘Servicios veterinarios de Sil’ veterinary clinic. The highest sales were recorded in 2018, and the lowest were in 2013. CanL vaccination began at the veterinary clinic in 2012, with the highest number of vaccinations recorded in that year, followed by 2022. The lowest recorded vaccination rate was in 2014. By 2022, a total of 816 CanL vaccines have been administered at the veterinary clinic.

**Table 4:** Global sales of ectoparasiticides and vaccinations against CanL at the ‘Servicios veterinarios de Sil’ veterinary clinic from 2003 to 2022

Year	No. ectoparasiticides	No. CanL vaccines
2003	145	0
2004	171	0
2005	154	0
2006	147	0
2007	159	0
2008	206	0
2009	185	0
2010	190	0
2011	191	0
2012	169	118
2013	132	85
2014	158	46
2015	135	51
2016	174	68
2017	200	71
2018	366	62
2019	268	83
2020	179	67
2021	181	72
2022	192	93
Total	3702	816

No. ectoparasiticides, global number of all types of ectoparasiticides sold; No. CanL Vaccines, total number of vaccines against CanL administered

**Fig. 2:**
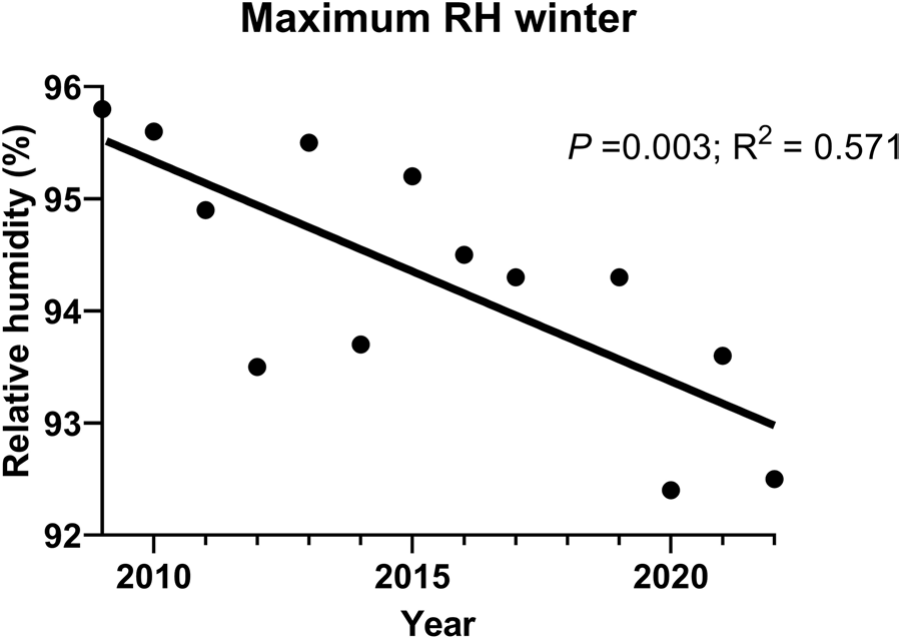
Maximum relative humidity (RH) in winter. Trend from 2009 to 2022. The line represents the linear trend (*P* = 0,003, *R*^*2*^ = 0.571), indicating a decreasing trend. Each data point corresponds to the mean maximum relative humidity in winter (December of the previous year to February of the following year)

The higher the maximum relative humidity in winter (Spearman's rank correlation coefficient (*rs*) = 0.60, *P* = 0.029), the greater the percentage of new *L. infantum* infection cases. This maximum relative humidity in winter showed a significant linear decline over time when data were available (Fig. 2).

Although not statistically significant, other climatic variables were correlated close to statistical significance with the percentage of positive dogs. This was the case for the maximum (*rs* = 0.39, *P* = 0.095) and the mean (*rs* = 0.42, *P* = 0.071) temperature in summer, which were positively correlated with the percentage of new positive cases.

With regard to prophylactic measures, higher sales of ectoparasiticides in the practice were correlated with a decrease in new *L. infantum* infection cases (*rs* = −0.56, *P* = 0.010). Similarly, there was a moderate negative correlation (*rs* = −0.52, *P* = 0.018) between the percentage of new *L. infantum* infection cases and vaccination against the disease in dogs.

## Discussion

To the best of our knowledge, this study, along with a recently published study in Spain [39], represents one of the most extensive longitudinal retrospective analyses of *L. infantum* serological status in owned dogs within our geographical area. Following the fundamental work of Amusategui et al. [13], which profoundly changed the understanding of *L. infantum* infection dynamics in Valdeorras and Ourense, subsequent investigations have explored the disease landscape in this region [5, 14, 16, 17].

The mean percentage of new seropositive cases to *L. infantum* infection identified over the 20-year duration of this study (21.65 ± 10.8%) aligns with the classification established by Gálvez et al. [5] of Ourense as a hyperendemic area (seroprevalence > 17%). In northern Spain, the seroprevalence of *L.infantum* infection is higher than in the rest of the Iberian Peninsula. Nonetheless, Ourense has been found to have high seroprevalences due to its highly suitable climatic conditions for *L. infantum* expansion [5]. The potential bias resulting from differences in methodology must be carefully considered when analysing the differences between this result and the seroprevalences obtained in previous studies [5, 13, 14, 17]. Some authors have used different *Leishmania* diagnostic tests (rapid test or ELISA) [14, 16], while others performed IFAT as done in this study [5, 13, 17]. It is important to consider that our study only used the first seropositive detection for each dog, whereas other studies determined the overall seroprevalence without making this distinction [5, 14, 16, 17].

Additionally, the number of positive dogs in our study may be different than in the overall community. On the one hand, considering the specific characteristics of a population attended in a veterinary clinic, this number can be higher due to a biased population of sick animals [40]. However, on the other hand, dogs attended in a veterinary practice usually have lower prevalences of the disease than other populations such as stray dogs, as previously shown in the same area of study [14].

The higher percentage of new seropositive cases observed during the initial lustrum (May 2003 to April 2008, 35.3%) may be attributed to the limited awareness and understanding of CanL among both pet owners and veterinarians within the region at that time. This lack of awareness might have resulted in a lower testing for the disease during routine health check-ups. Consequently, as previously shown in the area [14], fewer animals were being tested, and of those analysed, a greater proportion would have been found to be seropositive for *L. infantum* infection. As CanL gained greater attention within the veterinary community in this area, practitioners likely increased their focus on early diagnosis and prevention, as well as raising awareness among dog owners. This shift in approach could theoretically be one of the reasons that explains the decline in the number of new positive cases over time, despite the presence of potential favourable weather conditions for sand fly development. Similar explanations have been recently proposed for the declining prevalence of other vector-borne pathogens, such as *Dirofilaria immitis* [41].

Historically, dogs in rural areas of Spain, like Valdeorras, often lived outdoors, increasing their exposure to sand flies and, consequently, to *L. infantum* infection [14, 42–44]. However, contemporary trends indicate a shift towards dogs living indoors and experiencing improved lifestyles. This change, coupled with the implementation of enhanced preventive measures and disease management strategies, could also contribute to the observed decrease in the prevalence of infected dogs [43].

Regarding environmental factors, this study found a positive correlation between the maximum relative humidity during winter and new cases of *L. infantum* infection, corroborating the results of Risueño et al. [20]. They observed a positive association between maximum relative humidity from November to April and the presence of sand flies, which indirectly correlates with subsequent leishmaniosis cases [20]. This positive correlation could be attributed to the necessity of a humid environment for sand fly development [20, 45, 46]. However, it should be interpreted with caution since different variables acting simultaneously, such as the exact household of every dog and other climatic, ecological or sociological factors, may act as confounding factors.

The amount of rainwater could, on the one hand, promote the presence of organic matter (i.e. vegetation) essential for the vector’s biological cycle [20, 29, 47]. Conversely, the same rainfall might impede the sand fly’s flight. However, no correlation was found between recorded precipitation and the percentage of new cases of *L. infantum* infection in our study, which can be interpreted as a reflection of the lack of association between precipitation and sand fly presence observed in another study [7].

Temperature stands out as one of the most influential climatic factors affecting leishmaniosis transmission and sand fly ecology [7, 20, 37, 48]. However, different authors have reported conflicting results on its influence on sand fly presence, abundance or density and, consequently, in *L. infantum* transmission. Some studies have indicated that higher temperatures correlate with increased sand fly occurrence [7, 24, 26, 49, 50] and human leishmaniosis incidence [29, 37], while others have found no association or even a negative influence [47]. Variations in results could be attributed to factors such as the species of phlebotomine analysed, the specific bioclimatic area under study, or differences in the methods used to assess climatic data [8, 22, 47, 48, 51]. In our study, a certain trend towards significance was detected between an increase in maximum or mean temperature of the warmest season (summer) and an increased incidence of *L. infantum* infection. Although it has been established that sand flies remain active at a minimum temperature of 17 °C [28], our findings do not reveal a significant relationship between the lowest minimum temperature or the mean minimum temperature of the month of July (the month associated with the maximum risk of *L. infantum* transmission [28]) and the number of positive cases. The lowest minimum temperature recorded in July in Valdeorras consistently fell below 17 °C, with only two instances (2006 and 2022) showing a slight elevation in the mean minimum temperature. Nevertheless, it is worth noting that sand flies frequently seek refuge in burrows or shelters where temperatures may not dip as low [27, 28, 50].

Although this study has spanned a considerable length of time, its sample size (*n* = 20) precludes the possibility of conducting multivariate analysis with statistically significant outcomes. Thus, while the climatic findings are intriguing, a more extensive dataset may be necessary to accurately evaluate the impact of climate change. On the other hand, due to the retrospective nature of the study, we were unable to assess some other interesting climatic variables such as wind in this article.

These findings highlight the critical importance of preventive measures against canine leishmaniosis, particularly given the current trends in climatic variables potentially favouring the development of vector-borne disease. The results of the current study support this, considering the reduction in the proportion of newly reported positive cases in the area as annual sales of all types of ectoparasiticides and vaccination against *L. infantum* rates increase. In a recent article that examined preventive measures against *L. infantum* infection in dogs in Europe, repellents were the only measures that showed a small increase each year [44]. These results are consistent with the statement that one of the best preventive strategies against *L. infantum* in dogs is the use of external parasiticides that prevent phlebotomine sand flies from biting [2, 28, 32]. However, it is important to acknowledge certain limitations regarding the collection data in our study. It would have been interesting to have specific data about the use of repellents specifically registered for the prophylaxis of *L. infantum* infection. However, considering the long period of study and its retrospective nature, data were only accessible for overall sales in the clinic. Additionally, it is common for pet owners in our country to procure these products from external suppliers, potentially leading to even higher usage than what is recorded internally. The sale of ectoparasiticides for dogs at a specific veterinary clinic could reflect the proactive commitment of pet owners and practitioners to take care of their pets’ health, particularly in preventing parasitic diseases. The optimal prophylactic protocol for preventing *L. infantum* infection also involves the vaccination of healthy seronegative dogs in addition to the use of ectoparasiticides [2, 28, 32]. Vaccines currently available do not prevent the infection but diminish the risk of developing the disease [4, 28]. ‘Servicios veterinarios de Sil’ started vaccinating dogs against *L. infantum* in 2012, the year when the first vaccine was commercialised in Spain. In this context, one of the reasons for not including vaccinated dogs in our study was the description of cross-reactions between antibodies from vaccination and from natural infection, leading to potential misinterpretation of the IFAT [4]. Furthermore, by not including vaccinated dogs, some infected animals may have been overlooked.

Data from this study reflect the challenging situation of *L. infantum* infection during a very long period, in practice, in a specific area from Northwestern Spain, where the situation of the disease has been changing during the last decades. These results cannot be directly extrapolated to the general population. However, the extended follow-up period further emphasises the significance and importance of this study’s findings. By analysing data trends, veterinarians can gain valuable insights into the prevalence, distribution and progression of this disease in dogs. Furthermore, this study serves as a potential tool in informing veterinary practices and public health initiatives aimed at controlling canine leishmaniosis.

## Conclusions

To our knowledge, this is one of the longest studies evaluating the evolution of *L. infantum* infection in owned dogs in a fixed location and its association with external factors such as climatic conditions and preventive measures. The results obtained confirm that Valdeorras is a high-risk area for *L. infantum* infection. Although climatic conditions are fundamental in the ecology of the vector, they were not as relevant in the transmission of *L. infantum* as the use of ectoparasiticides and vaccines against *L. infantum* infection. Clinicians need to be aware of *L. infantum* infection, its aetiopathogenesis and strategies to control the disease. The importance of veterinarians raising awareness among dog owners of the seriousness of the disease and the need for preventive measures to protect both pets and public health is a cornerstone in the management of this vector-borne parasitic disease.

## Data Availability

All data regarding this study are included in the manuscript.

## Abbreviations

CanL: Canine leishmaniosis
IFAT: Indirect immunofluorescence antibody tests
IgG: Immunoglobulin G
